# Children's vomiting following posterior fossa surgery: A retrospective study

**DOI:** 10.1186/1472-6955-8-7

**Published:** 2009-07-13

**Authors:** Susan M Neufeld, Christine V Newburn-Cook, Donald Schopflocher, Belinda Dundon, Herta Yu, Jane E Drummond

**Affiliations:** 1 Faculty of Nursing, University of Alberta, Edmonton, Canada; 2 Hospital for Sick Children, Toronto, Ontario, Canada

## Abstract

**Background:**

Nausea and vomiting is a problem for children after neurosurgery and those requiring posterior fossa procedures appear to have a high incidence. This clinical observation has not been quantified nor have risk factors unique to this group of children been elucidated.

**Methods:**

A six year retrospective chart audit at two Canadian children's hospitals was conducted. The incidence of nausea and vomiting was extracted. Hierarchical multivariable logistic regression was used to quantify risk and protective factors at 120 hours after surgery and early vs. late vomiting.

**Results:**

The incidence of vomiting over a ten day postoperative period was 76.7%. Documented vomiting ranged from single events to greater than 20 over the same period. In the final multivariable model: adolescents (age 12 to <17) were less likely to vomit by 120 hours after surgery than other age groups; those who received desflurane, when compared to all other volatile anesthetics, were more likely to vomit, yet the use of ondansetron with desflurane decre kelihood. Children who had intraoperative ondansetron were more likely to vomit in the final multivariable model (perhaps because of its use, in the clinical judgment of the anesthesiologist, for children considered at risk). Children who started vomiting in the first 24 hours were more likely to be school age (groups 4 to <7 and 7 to <12) and receive desflurane. Nausea was not well documented and was therefore not analyzed.

**Conclusion:**

The incidence of vomiting in children after posterior fossa surgery is sufficient to consider all children requiring these procedures to be at high risk for POV. Nausea requires better assessment and documentation.

## Background

The successful management of nausea and vomiting is an important component in the care of children after surgery. Postoperative nausea and vomiting (PONV) may cause discomfort and distress, put pressure on surgical incisions, cause dehydration and electrolyte imbalance, delay recovery, and prolong hospitalization [1,2]. Children are at high risk for experiencing PONV [3-5], and estimates of postoperative vomiting (POV) for children requiring craniotomy have been as high as 66% [6]. The effects of increased intracranial pressure during retching and vomiting may be especially problematic after craniotomy [7]. Therefore, children after craniotomy may be at particular risk for experiencing PONV that result in suffering and other negative outcomes.

Tramer [8] describes three rules of practice to ensure optimal management of PONV: identify those at risk using predictive factors; modify anaesthesia techniques to keep the baseline risk as low as possible; and administer anti-emetics rationally with consideration to their efficacy, additive properties, and adverse effects. Of these, Tramer concedes that knowledge of risk factors remains an under-researched area, especially for children. In one of the few multivariable studies of predictive factors of POV in children [9], the combination of the following factors could be used to determine a child's risk of POV after surgery: history of POV in the child or PONV in father, mother or siblings; age over three years; length of surgery over thirty minutes; and strabismus surgery. The results of this study were further validated in a separate group of children [10] and were predictive of POV even when strabismus surgery was not in the prognostic model. These studies did not have the participation of children requiring craniotomy. However, if the prognostic models were used to predict POV in children after craniotomy, age and history of POV or PONV in father, mother, or siblings would be the only variables that could be used to predict POV.

POV and/or PONV after craniotomy in children have not been well described in the literature. Two small randomized controlled trials of ondansetron, a 5-HT_3 _receptor antagonist, have estimates of 24 hour POV in children after craniotomy that range from 24% [11] to 66% [6], whereas PONV in older children was estimated at 32% [11]. Although some clinicians believe that the use of prophylactic anti-emetics decreases the incidence of POV in these children, neither study could show efficacy for intraoperative administration of ondansetron in reducing children's POV by 24 hours. Subramaniam et al. [10] could not show evidence for an extra scheduled postoperative dose of ondansetron either. Finally, intermittent dosing of any class of anti-emetics does not appear to have been studied in this patient population. In order to effectively design such studies, knowledge of the incidence of PONV and associated risk and protective factors must be first established to know the extent of the problem and ensure that the children at highest risk are targeted for prophylaxis.

Children who appear to be at high risk for nausea and vomiting are those who require posterior fossa craniotomy. Posterior fossa surgery takes place below the tentorium cerebelli in the posterior cranial fossa. The posterior cranial fossa houses structures that include: the cerebellum; brainstem; and cranial nerves III-XII. Although the reticulospinal tracts, diencephalon, limbic system, and discrete areas of the cerebral hemispheres may all be involved in nausea, retching, and vomiting, the coordination of the autonomic changes associated with retching and vomiting occurs at the level of the medulla oblongata in the posterior fossa [12-14]. Thus, from an anatomical perspective, procedures that are proximal to this area may place patients at especially high risk for vomiting. The aforementioned studies in children after craniotomy [6,11] have been too small to allow this conclusion to be drawn, but studies in adults suggest that posterior fossa procedures are related to greater postoperative nausea (PON) [7] and PONV [15-17] when compared to supratentorial procedures. However, Quiney and colleagues [18] prospectively examined nausea and vomiting in 52 adults and children (aged 8–74) after craniotomy and found that 37% of participants experienced severe nausea or vomiting, while 35% experienced nausea at the end of the 24 hour period. These authors could not find any relationship between location of surgery and symptoms; however, their small sample size limits the validity of this finding.

Children who require craniotomy form a heterogeneous group. By specifically studying children after posterior fossa surgery, the research questions can be focused on the unique risk and protective factors for PONV for this group of children, while limiting the heterogeneity in the sample. This approach has shown success in determining risk and protective factors specific to the location of the neurosurgery in adult studies [19-21]. Thus, the purpose of this study was to describe PONV in children requiring posterior fossa surgery, to explore risk and protective factors for PONV, and to examine the relationship of PONV to adverse outcomes. Results could then be used to guide the design of future studies and provide the rationale for implementing improvements to clinical practice.

## Methods

The hospital charts of children who required posterior fossa surgery at two Canadian children's hospital sites, the Stollery Children's Hospital in Edmonton and the Hospital for Sick Children in Toronto, were reviewed for the study. A retrospective study design was chosen as an efficient and cost effective way to describe nausea and vomiting in this group of children. Specifically, for the acute postoperative period, we sought to determine:

1) The cumulative incidence of nausea and vomiting by: hours 4, 8, and 24; and during subsequent 24 hour periods.

2) The frequency distribution of number of days that nausea and vomiting were experienced.

3) The frequency distribution of number of vomiting events.

4) The significant risk and protective factors for nausea and vomiting.

5) The co-morbidities that children with nausea and vomiting experience.

### Sample Selection & Sample Size Estimation

Following institutional ethical review and administrative approval at each centre, all patients under age seventeen who had posterior fossa surgery between March 1, 2001 and March 1, 2007 were identified for chart retrieval through two separate paediatric neurosurgeons' databases (Stollery Children's Hospital) and a central paediatric neurosurgery database (Hospital for Sick Children). The upper limit for age was determined by the age of qualification for admission to both children's hospitals. Fourth ventricular shunt procedures, operations without dural openings (outside the brain), surgery for traumatic brain injury, and children requiring prolonged intubation (greater than 48 hours) were excluded. If more than one posterior fossa procedure was required for a child over the study period, the earliest one was included in the study. Prospectively, we estimated data from two sites to involve approximately 300 children. This would allow for an estimation of incidence within a 6% margin of error [22]. It would also allow for a multivariable analysis of 10–15 variables if the incidence of an outcome ranged from 30–70%, which would allow for one independent variable per ten outcomes [23].

### Data Collection Procedures

A case report form was developed specifically for the study in collaboration with a paediatric educator in surgery, a neurosurgeon, and clinical nurse specialists/advanced nurse practitioners in children's neurosurgery. Data were collected by review of the child's in-patient chart. The data collection period extended over the course of a child's neurosurgical postoperative hospital stay, up to ten days. Thus, data collection ended when the child went home, was transferred from neurosurgical care to rehabilitation care (i.e. to a rehabilitation hospital or rehabilitation unit), was transferred from neurosurgery care to oncology care for further treatments, or at 240 hours after the recorded time that the anaesthetic was finished. The first ten postoperative days were chosen by the study team to ensure that an adequate length of time was captured for the exploratory analysis. The final outcomes for analysis of risk and protective factors were determined based on these exploratory results.

One nurse with paediatric neurosurgical experience at each site collected data. To ensure reliability, the two data collectors trained on ten charts. Revisions to the data collection form were then made as necessary. For example, the child's activity and diet at each time period were initially part of the case report form. Due to gaps in charting, these data could not be collected reliably. Once the case report was finalized, each person then reviewed the same five randomly selected charts to establish inter-rater reliability: 100% inter-rater reliability was achieved for the main study outcomes, independent variables and adverse events. Weekly contact was maintained between the two sites to discuss issues that arose during data collection.

### Measurement

PONV covers one or more of three symptoms: nausea; retching; and vomiting [24]. Nausea is the unpleasant sensation of the urge to vomit that occurs along with neurological changes such as excessive salivation and swallowing [25]. Each time nausea was charted in the nurses' notes, it was recorded on the study case report form. Documented children's statements or behaviours that referred to nausea, such as states that he feels "like throwing up" or "appears nauseous," were also included.

Retching is the first phase of vomiting [12] and is commonly defined as an unproductive attempt to vomit [25]. Vomiting is the forceful expulsion of stomach contents through the mouth that involves coordinated autonomic processes in the brain and gut [12]. Because of their similar physiology, retching and vomiting should be considered together in the data analysis, whereas nausea should be considered separately [26]. To screen for retching and vomiting events (POV), the post-anaesthesia recovery room record was first reviewed, followed by the in and out flow sheets. The time of the event was noted. The nurses' notes and assessment flow charts were then read for further events, for more accurate time of the event if it corresponded to the in-and-out flow sheets, and to screen for retching episodes. If retching and vomiting were documented at the same time, they were considered one event. The medication administration records were reviewed and anti-emetic administration was noted. If an anti-emetic was administered, the nurses' notes were reviewed a second time to look for a documented retching or vomiting event around the time of the administration of the anti-emetic. Administration of an anti-emetic was not considered indication of nausea, retching or vomiting.

Data on potential risk factors were collected from the admission records, physician notes, anaesthesia records, operative reports, and medication flow sheets. This data included age of child, gender, presenting symptoms, type of surgery, length of surgery, use of intraoperative dexamethasone and/or ondansetron (administration at any time after induction), and type of anaesthetic. For analysis, age was examined in quartiles rounded to the year: 0 to <4; 4 to <7; 7 to <12; and 12 to <17. A number of variables were dichotomized for analysis. The use of desflurane has been shown to be a risk factor in adults requiring retromastoid craniectomy, compared to other volatile anaesthetics [20]. We therefore examined the use of desflurane (alone or in combination with another volatile anaesthetic) compared to a grouping of all other volatile anaesthetics. This was the only variable with missing values (n = 13; either undocumented in the chart or illegible) and, due to the likelihood that the missing value was not desflurane, we included the missing values in the "other" category (the mode was isoflurane). The use of nitrous oxide, a well known risk factor for PONV [24], was rarely documented for induction and not used for maintenance, and therefore was not assessed as in intraoperative risk factor. Intra-operatively, ondansetron was the only anti-emetic used, and dexamethasone the only steroid.

Finally, Potential co-morbidities, including development of a pseudomeningocele, wound failure or cerebral spinal fluid leak through the incision, and wound infection, were noted by examining the nursing notes, physician notes, discharge summary, and/or reasons for readmission to the neurosurgical service. Reports of postoperative imaging studies were also reviewed for possible documentation of a pseudomeningocele.

### Data Analysis

Data analysis was conducted using SPSS Version 15.0 software. Demographic and study variables were summarized using descriptive statistics that were appropriate to their level of measurement. Cumulative incidence (number of children with at least one recorded event by the specified time period/total number of children in the study) was used to calculate outcomes at: 4, 8, and 24 hours, and for subsequent 24 hour periods until the end of the study period. The number of days that an outcome was experienced was calculated by subtracting the time of the first recorded event from the last recorded event, and was summarized using a frequency distribution. A frequency distribution was also used for counts of recorded events.

Univariate logistic regression was first conducted to examine the relationships between each variable and the outcomes. Additionally, the clinical plausibility of an interaction effect of ondansetron and dexamethasone was tested using two steps. In the first step, the adjusted odds ratios of ondansetron and dexamethasone were estimated using multivariable logistic regression. Then, the interaction term of ondansetron and dexamethasone was entered in the second step.

**Figure 1:**
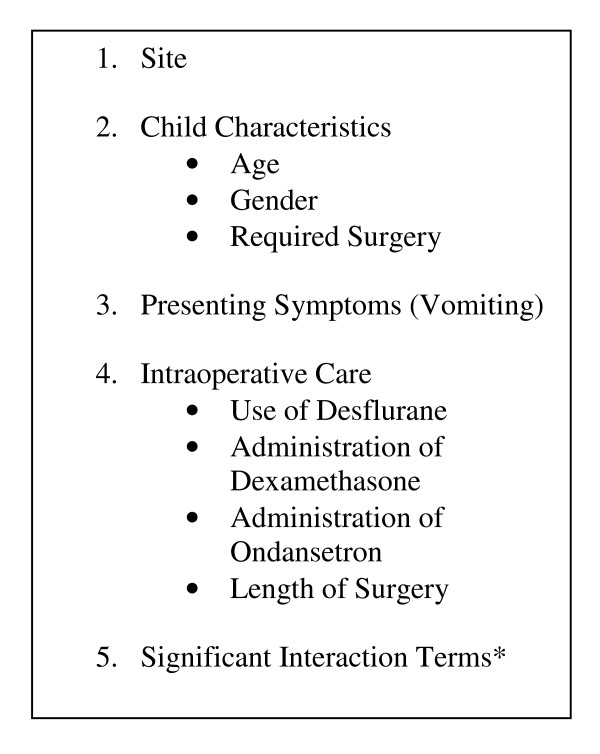
**Hierarchical model for variable entry into multivariable analysis**. *Interaction terms tested will be intraoperative ondansetron and dexamethasone as well as combinations of plausible statistically significant variables from the multivariable model.

The univariate analysis was followed by multivariable logistic regression to examine important risk and protective factors for the outcomes while controlling for other variables. To determine confounding effects, variables were entered into the multivariable model in an *a-priori *determined, hierarchical fashion based on sequential events (Figure 1). If a variable grouping changed the regression coefficients of a previously entered statistically significant variable by ≥15%, it was considered to be confounding (i.e., related to both the variable and the outcome). Conversely, if a variable that was not previously statistically significant became so with a change of ≥15%, that grouping was also considered confounding. Individual variables within that grouping were then tested individually to examine their relationship to the confounded variable.

In the final step, plausible interactions of statistically significant variables were tested to determine if any moderating effects were present (i.e., that the relationship that one variable has with an outcome changes depending on the value of another variable). The clinical plausibility of dexamethasone and ondansetron administered together vs. either drug administered alone were again tested using an interaction term in the larger multivariable models at this time. Only statistically significant interactions were included in the final model. Multicollinearity, associations among the independent variables in the model, was reviewed by looking for correlations above 0.8 between any two variables.

To finish the analysis, cross tabulations were used to examine the relationship between the final outcomes and other adverse events. The phi statistic (ϕ) for nominal by nominal variables was used to summarize these relationships. This statistic was also used to examine relationships between categorical confounding variables, whereas a Pearson's correlation (r) was used for continuous variables.

## Results

### Sample Characteristics

Table 1 contains a description of the study sample. A total of 249 children met the criteria for the study, from 329 potential candidates who were identified from three neurosurgery databases. Of those excluded, 23 were wrongly coded as posterior fossa procedures in the database, 22 were intubated for more than 48 hours, 13 had Chiari I bony decompressions without a dural opening, 13 had a supratentorial component to their surgery (other than EVD/shunt insertion), 7 had ventriculo-peritoneal shunt procedures only, and 2 had no corresponding hospital record.

**Table 1:** Sample characteristics

Parameter	
Number of Patients	249

Site n(%)	

Stollery Children's Hospital	55 (22.1%)

Hospital for Sick Children	194 (77.9%)

Length of hospital stay in days (Mean ± SD, Range)	11 (± 21, 2–305)

Age in years (Mean ± SD, Range)	7.6 ± 4.4, 0.3–-16.8

Age in Quartiles n(%)	

0 to <4 years	65 (26.1%)

4 to <7 years	61 (24.5%)

7 to < 12 years	75 (30.1%)

12 to <17 years	48 (19.3%)

Male: Female (Ratio %)	128 (51.4%):121 (48.6%)

Surgery n(%)	

Brain Tumour	153 (61.4%)

Chiari I Malformation	81 (32.5%)

Other	15 (6.0%)

Chiari II Malformation	7 (2.8%)

Vascular Malformation	4 (1.6%)

Cyst or Aspiration of Pus	4 (1.6%)

Presenting with vomiting n(%)	115 (46.2%)

Preoperative Dexamethasone n(%)^1^	136 (54.6%)

Intraoperative Ondansetron	117 (47.0%)
Without Dexamethasone	47 (18.9%)

Intraoperative Dexamethasone	131 (52.6%)
Without Ondansetron	61 (24.5%)

Intraoperative Ondansetron and Dexamethasone	70 (28.1%)

No intraoperative anti-emetic	71 (28.5%)

Length of surgery in hours (Mean ± SD, Range)	5:01 ± 2:10 (1:39–17:56)

Length of anaesthesia in hours (Mean ± SD, Range)	6:22 ± 2:20 (2:05–20:15)

Type of volatile anaesthetic n(%)	

Isoflurane	126 (50.6%)

Desflurane	46 (18.5%)

Isoflurane + Sevoflurane	32 (12.9%)

Sevoflurane	22 (8.8%)

Isoflurane + Desflurane	8 (3.2%)

Sevoflurane + Desflurane	2 (0.8%)

Not recorded/not legible	13 (5.2%)

Use of Nitrous Oxide**	7 (2.8%)

Received Postoperative Anti-emetic (%)	199 (79.9%)

Opioid administration initiated by the first 24 hours (%)	228 (91.6%)

*All but one child who received preoperative dexamethasone had a brain tumour.

**For induction only as no nitrous oxide was used for maintenance of anaesthesia.

### Description of PON, POV and PONV

**Figure 2:**
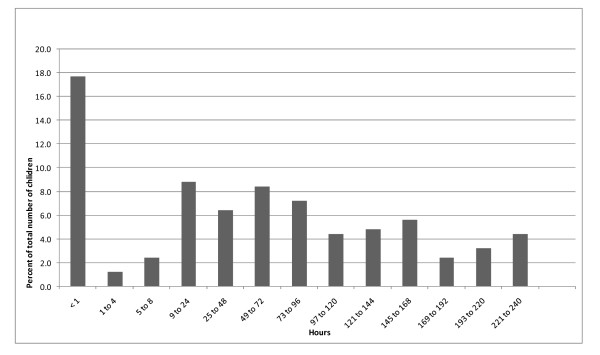
**Hours from first recorded retching or vomiting to last in the 240 hours**.

**Figure 3:**
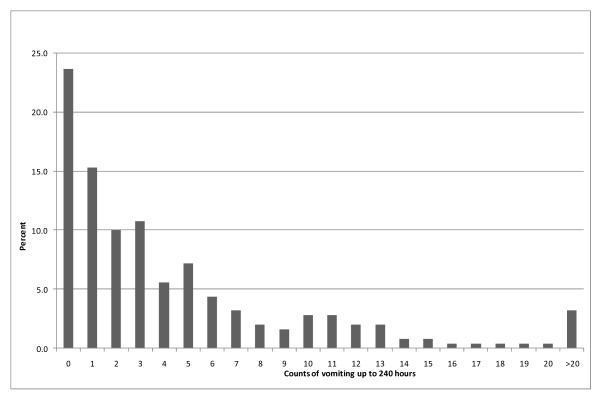
**Number of recorded retching or vomiting events over the study period**.

The cumulative incidence of PON, POV and PONV over the first ten days is presented in Table 2. As shown in this table, there was a discrepancy in the documentation of PON and POV. Because we felt that PON was not reliably measured and documented, the remainder of the data analysis was refocused to examine POV. The frequency distribution of the time from first recorded POV to last recorded POV is presented in Figure 2. The frequency distribution of POV events that were recorded over the study period is shown in Figure 3. These figures indicate that there was considerable variation in length of time that children experienced vomiting as well as number of recorded events. Close to 47% of children experienced vomiting over a time course greater than 24 hours, while 20% continued to vomit over a time course greater than 120 hours. Recorded events, shown in Figure 3, show a positively skewed distribution with 23% children with no events, 36% of children with only one to three recorded events, and 41% with over three events.

**Table 2:** Cumulative incidence of retching/vomiting, nausea and PONV

	Hours from anaesthetic finish time Total number of children with least one event recorded (Cumulative Percent)										
	
	0–4	0–8	0–24	0–48	0–72	0–96	0–120	0–144	0–168	0–192	0–216*
Retching/Vomiting	59 (23.7%)	75 (30.1%)	119 (47.8%)	153 (61.4%)	164 (65.9%)	174 (69.9%)	181 (72.7%)	186 (74.7%)	187 (75.1%)	190 (76.3%)	191 (76.7%)

Nausea **	24 (9.6%)	32 (12.9%)	59 (23.7%)	84 (33.7%)	93 (37.3%)	98 (39.5%)	103 (41.4%)	105 (42.2%)	105 (42.2%)	106 (42.6%)	107 (43.0%)

PONV	69 (27.7%)	81 (32.5%)	134 (53.8%)	162 (65.1%)	172 (69.1%)	182 (73.1%)	189 (75.9%)	190 (76.3%)	191 (76.7)	194 (77.9%)	195 (78.3%)

PONV: First recorded nausea, retching or vomiting.

*No new recorded events after 216 hours.

**The differences between the incidence of nausea and retching/vomiting indicate that nausea is underestimated.

### Analysis of risk and protective factors

Based on the initial exploratory data analysis, we decided to examine the risk and/or protective factors for two outcomes: POV in the first 120 hours (the acute postoperative period), and early (an event recorded by 24 hours or less) compared to late POV (the first event occurring after 24 hours up to 120 hours). The decision to make the cut-off for the acute postoperative period 120 hours, despite data collection that went up to 240 hours, was to control for confounding factors such as early discharge, the requirement for sedation for procedures or tests, or the need for further surgery that were emerging in the data after 120 hours. After data collection, it was clear that some potential risk factors could not be reliably collected due to lack of documentation or inconsistent documentation. These included: a history of PONV in the child or family member; a history or motion sickness; and pain rating.

The initial univariate logistic regressions (Table 3) indicated that only Chiari I Malformation surgery was a statistically significant risk factor for POV by 120 hours. Additionally, children in the two middle age quartiles (4 to <7 and 7 to <12) showed lower odds of late vomiting compared to early. Thus, while overall these children did not have greater odds of vomiting, they were more likely to have early vomiting than the other age groups. When testing for an interaction effect for ondansetron and dexamethasone, the first step showed that, when controlling for dexamethasone, ondansetron became positively predictive of POV by 120 hours. The second step showed that the interaction effect (i.e. an enhanced effect of both drugs together) of ondansetron and dexamethasone was not significant.

**Table 3:** Univariate analysis

Variable	POV by 120 Hours Odds Ratio (95% CI)	Early (≤ 24 hours) vs. Late POV^1^ Odds Ratio (95% CI)
1. Site		

Stollery Children's Hospital	1.00	1.00

Hospital for Sick Children	1.74 (0.92–3.29)	1.71 (0.73–4.03)

2. Child Characteristics		

Age		

0 to <4 years	1.00	1.00

4 to <6 years	1.10 (0.48–2.49)	0.26 (0.11–0.64)**

7 to <12 years	0.90 (0.42–1.92)	0.30 (0.13–0.69)**

12 to <17 years	0.54 (0.24–1.23)	0.41 (0.16–1.08)

Female Gender	0.79 (0.45–1.38)	1.39 (0.74– 2.62)

Required Surgery		

Brain Tumour	1.00	1.00

Chiari I Malformation	2.32 (1.17–4.60)*	0.60 (0.30–1.19)

Other	0.39 (0.13–1.13)	4.86 (0.90–26.30)

3. Presenting Symptom		

Presenting with Vomiting	0.95(0.55–1.67)	1.09 (0.58–2.05)

4. Intraoperative Care		

Administration of Ondansetron	1.78 (1.00–3.15)	0.74 (0.39–1.38)

Administration of Dexamethasone	0.65 (0.37–1.15)	1.62 (0.86–3.05)

Length of surgery	1.00 (0.98–1.01)	1.00 (0.98–1.01)

Use of Desflurane	1.32 (0.66–2.64)	0.34 (0.14–0.83)*

5. Test of Interaction: Ondansetron and Dexamethasone	Adjusted Odds Ratio (95% CI)	Adjusted Odds Ratio (95% CI)

Step 1		

Intraoperative ondansetron	1.92 (1.07–3.45)*	1.75 (1.91–3.35)

Intraoperative dexamethasone	0.59 (0.33–1.06)	0.66 (0.34–1.26)

Step 2		

Intraoperative ondansetron	1.43 (0.58–3.53)	0.37 (0.13–1.03)

Intraoperative dexamethasone	0.49 (0.23–1.02)	1.10 (0.45–2.67)

Dexamethasone X ondansetron	1.63 (0.50–5.30)	2.86 (0.73–11.13)

1. Early vomiting coded as 0 and late as 1, no POV counted as missing therefore n = 181.

*p < .05

**p < .01

CI = Confidence Interval

In the first multivariable analysis (Table 4), controlling for the other variables in the model, children with any use of desflurane (compared to the grouping of children who received either sevoflurane, isoflurane, or a combination of these), who received intraoperative ondansetron, who required Chiari I malformation surgery or who had surgery at the Hospital for Sick Children site had an increased likelihood of vomiting by 120 hours. Conversely, children in the fourth age quartile (12 to <17) were less likely to vomit by 120 hours. Finally, the significant interaction term indicated that intraoperative ondansetron moderated the likelihood of vomiting with desflurane by 120 hours. Thus, those children who received desflurane were less likely to vomit if given intraoperative ondansetron than were those who received desflurane without receiving intraoperative ondansetron.

**Table 4:** Multivariable analysis: POV by 120 hours

Model Number	5	4	3	2	1
Variable	Adjusted Odds Ratio (95% CI)	Adjusted Odds Ratio (95% CI)	Adjusted Odds Ratio (95% CI)	Adjusted Odds Ratio (95% CI)	Odds Ratio (95% CI)

1. Site					

Stollery Children's Hospital	1.00	1.00	1.00	1.00	1.00

Hospital for Sick Children	3.92 (1.45–10.58)*	4.25 (1.62–11.12)^α^**	1.78 (0.90–3.51)	1.78 (0.90–3.50)	1.74 (0.92–3.29)

2. Child Characteristics					

Age in quartiles%					

0 to <4 years	1.00	1.00	1.00	1.00	

4 to <7 years	0.86 (0.35–2.14)	0.84 (0.34–2.06)	0.96 (0.41–2.27)	1.00 (0.43–2.35)	

7 to <12 years	0.51 (0.21–1.22)	0.56 (0.24–1.31)	0.72 (0.32–1.61)	0.74 (0.33–1.64)	

12 to <17 years	0.34 (0.13–0.88)*	0.33 (0.13–0.82)^α^*	0.48 (0.20–1.12)	0.47 (0.20–1.11)	

Female Gender	0.64 (0.34–2.14)	0.66 (0.35–1.21)	0.71 (0.39–1.27)	0.69 (0.39–1.25)	

Surgery					

Brain Tumour	1.00	1.00	1.00	1.00	

Chiari I Malformation	2.78 (1.03–7.50)*	2.43 (0.91–6.48)^α^	3.46 (1.48–8.10)^α^**	2.80 (1.37–5.71)**	

Other	0.65 (0.31–1.35)	0.53 (0.14–1.99)	0.54 (0.17–1.79)	0.44 (0.15–1.35)	

3. Presenting Symptoms					

Presenting with Vomiting	1.75 (0.79–3.91)	1.63 (0.75–3.54)	1.40 (0.67–2.90)		

4. Intraoperative Care					

Administration of Ondansetron	3.47 (1.61–7.47)**	2.22 (1.14–4.33)*			

Administration of Dexamethasone	0.65 (0.31–1.35)	0.58 (0.28–1.19)			

Length of surgery	0.90 (.76–1.06)	0.91 (0.78–1.08)			

Use of Desflurane	14.08 (2.47–80.32)**	3.11 (1.13–8.62)*			

5. Significant Interaction					

Use of Desflurane X Intraoperative administration of ondansetron	0.10 (0.12–0.55)**				

*p < .05

**p < .01

α Change ≥ 15% in a statistically significant variable or ≥ 15% making a variable statistically significant

CI = Confidence Interval

There were a number of variables which appeared to confound the effects of previously entered variables in the model. When the factor of whether or not a child presented with vomiting was added (Model 3), the odds ratio for POV by 120 hours for Chiari I surgery increased. A greater proportion of children with brain tumours presented with vomiting than those with Chiari I malformations or other procedures (72% vs. 5% vs. 7%, phi statistic for correlation of nominal variables (ϕ) = .0.65, p < .001); thus, controlling for whether or not the child presented with vomiting proportionally decreased the odds of vomiting for children with brain tumours compared to those with Chiari I malformations. The combined surgery category of "other" was too small (n = 15) to determine if there was similar effect. The odds ratio for hospital site changed to being significantly greater for the Hospital for Sick Children after the intraoperative variables were entered (Model 4). Desflurane and intraoperative ondansetron were used proportionately more at the Stollery Children's Hospital than at the Hospital for Sick Children (69% vs. 9% for use of desflurane, ϕ = 0.59, p < .001 and 62% vs. 43% for ondansetron, ϕ = -0.16, p = .01). There were no differences between the sites for the use of dexamethasone (ϕ = 0.08, p = .23) or length of surgery (r = -0.02 p = .74). Thus, controlling for the combination of intraoperative risk factors, notably any use of desflurane and administration of intraoperative ondansetron, resulted in a proportionately higher reduction in the risk of vomiting by 120 hours at the Stollery Children's Hospital compared to the Hospital for Sick Children. These findings highlight the challenges that can be found in types of procedures and/or sites with different proportions of children exposed to potential risk and protective factors.

Finally, the addition of the interaction term of any use of desflurane and the administration of ondansetron (Model 5) also showed a change in the odds of POV by 120 hours for children requiring Chiari I surgery to a statistically significant result. However, there were no differences in the proportion of children in the three surgery categories who received both ondansetron and desflurane (13% vs. 17% vs. 13%, ϕ = .06, p = .68). The interaction of intraoperative ondansetron and dexamethasone was not statistically significant when tested with this multivariable model.

The second multivariable modelling procedure (Table 5) was used to look at early (within 24 hours of their anaesthetic finish time) vs. late vomiting (after the first 24 hours up to 120 hours. The results of this analysis indicate that, controlling for the other variables in the model, children who received desflurane were more likely to begin vomiting within 24 hours of their anaesthetic finish time (early vomiting) than those who received one in the grouping of other volatile anaesthetics. Those in the two middle age quartiles (ages 4 to <7 and 7 to <12) were also more likely to start vomiting in the first 24 hours compared to those under age 4 and 12 to <17. There was no significant interaction between intraoperative administration of ondansetron and dexamethasone in this model either.

**Table 5:** Multivariable analysis: Early vs. late POV (POV in the first 24 hours vs. after 24 hours to 120 hours)^1^

Model Number	4	3	2	1
Variable	Adjusted Odds Ratio (95% CI)	Adjusted Odds Ratio (95% CI)	Adjusted Odds Ratio (95% CI)	Odds Ratio (95% CI)

1. Site				

Stollery Children's Hospital	1.00	1.00	1.00	1.00

Hospital for Sick Children	0.72 (0.22–2.19)	1.80 (0.72–4.52)	1.80 (0.72–4.50)	1.71 (0.72–4.03)

2. Child Characteristics				

Age in quartiles%				

0 to <4 years	1.00	1.00	1.00	

4 to <7 years	0.30 (0.11–0.79) *	0.31 (0.12–0.79)*	0.31 (0.12–0.78)*	

7 to <12 years	0.31 (0.13–0.79)*	0.32 (0.13–0.77)*	0.32 (0.13–0.76)*	

12 to <17 years	0.50 (0.17–1.48)	0.47 (0.17–1.30)	0.47 (0.17–1.29)	

Female Gender	1.90 (0.93–3.91)	1.62 (0.81–3.21)	1.62 (0.82–3.21)	

Required Surgery				

Brain Tumour	1.00	1.00	1.00	

Chiari I Malformation	0.64 (0.22–1.88)	0.64 (0.20–1.38)	0.57 (0.27–1.20)	

Other	2.82 (0.39–20.70)	2.63 (0.40–16.19)	2.70 (0.45–16.14)	

3. Presenting Symptom				

Presenting with Vomiting	0.71 (0.26–1.84)	0.89 (0.36–2.17)		

4. Intraoperative Care				

Administration of Ondansetron	0.85 (0.40–1.80)			

Administration of Dexamethasone	2.02 (0.87–4.68)			

Length of surgery	1.00 (0.98–1.02)			

Use of Desflurane	0.23 (0.07–0.73)*			

1. Early vomiting coded as 0 and late vomiting coded as 1, n = 181.

*p < .05 **p < .01

α Change ≥ 15% in a statistically significant variable or ≥ 15% making a variable statistically significant CI = Confidence Interval

Assessment for multicollinearity (Table 6), showed no estimates with correlations above .80. These results are suggestive that multicollinearity may not play a large role in the statistical model. The statistically significant correlations in this part of the statistical analysis correspond to the results already identified in the assessment for confounding effects (i.e., variables in a model that are related to each other and the outcome).

**Table 6:** Correlations among independent variables in the model

Variable		1	2	3	4	5	6	7	8	9	10	11	12
Site	1	1.00											

4 to <6 years^1^	2	-.06	1.00										

7 to <12 years^1^	3	.16	-	1.00									

12 to <17 years^1^	4	-.08	-	-	1.00								

Female	5	-.03	-.03	.03	.03	1.00							

Chiari I Malformation^2^	6	-.04	-.02	.01	.10	.15	1.00						

Other^2^	7	-.07	-.07	-.09	.09	-.01	-	1.00					

Presenting with Vomiting	8	.05	.11	.04	-.15	-.13	-.57*	-.20*	1.00				

Administration of Ondansetron	9	-.16	.01	.10	.11	.06	.03	-.10	.03	1.00			

Administration of Dexamethasone	10	.08	.09	-.01	-.09	-.03	-.46*	-.10	.40*	.14	1.00		

Length of surgery	11	.08	.09	-.01	-.09	-.02	.45*	-.10	-.39*	.14	.30*	1.00	

Use of Desflurane	12	-.59	.01	-.12	-.13	.07	.04	-.02	-.08	.19	.03	.00	1.00

1. Reference category 0–<4

2. Reference category brain tumour surgery *Two tailed p < .01

### Co-morbidities

With the exception of infection, adverse events were frequently reported in the sample. Because posterior fossa syndrome is most commonly associated with brain tumours, the relationship between posterior fossa syndrome and POV by 120 hours was examined for brain tumours only. As shown in Table 7, there was no relationship between POV by 120 hours and the development of pseudomeningocele, wound failure or cerebral spinal fluid leak, infection, or posterior fossa syndrome.

**Table 7:** Relationship of POV by 120 hours to adverse outcomes

	Vomiting by 120 hours n (% total)		Phi Statistic (Significance)
Total (n = 249)	No	Yes	

Pseudomeningocele			
No	49 (19.7)	124 (49.8)	
Yes	19 (7.6)	57 (22.9)	0.03 (p = .59)

Would Failure/CSF Leak			
No	55 (22.1)	158 (63.5)	
Yes	13(5.2)	23 (9.2)	-0.08 (p = .20)

Wound Infection			
No	63 (25.3)	175 (70.3)	
Yes	5 (2.0)	6 (2.4)	-0.09 (p = .17)

Brain Tumour (n = 153)			

Posterior Fossa Syndrome			
No	34 (22.2)	82 (53.6)	
Yes	13 (8.5)	24 (15.7)	-0.05 (p = .50)

## Discussion

The results of this study support our clinical experience that POV is a common adverse outcome for children after posterior fossa surgery. Overall, POV is common enough to regard all children who require posterior fossa surgery as being at high risk for the development of POV. When POV occurred, counts of vomiting events formed a positively skewed distribution in this sample. These results are similar to those shown by the data collected by Rowley and Brown [3] in their classic post-operative vomiting study of 1183 children after surgery. Many children in their sample experienced one or two episodes, with the number of events that a child experienced quickly tapering off. Rowley and Brown urged researchers to identify the recurrent, frequent, and distressing vomiting that fewer children experience but which results in significant distress and negative consequences for recovery. In this study, many children experienced greater than three events and/or experienced vomiting for time periods much longer than twenty four hours. This finding also has to be in the context of the use of intra-operative ondansetron for 47% of the children and use of postoperative anti-emetics in 80% of the children. Thus, even with current efforts to prevent and treat POV, it was a frequent, recurrent and potentially long-lasting problem in this sample of children.

In this study, some variables were related to POV in children after posterior fossa surgery. These results may lead to the development of predictive tools or the identification of areas for stratification for future research. They will also help to identify challenges for future research, especially multi-site studies. That one hospital site showed greater odds of POV by 120 hours, once intraoperative variables were entered, highlights the difficulty in comparing between sites that may have varying intraoperative practices. Type of surgery was significant, with children requiring surgery for Chiari I Malformation more likely to have POV by 120 hours than children requiring brain tumour craniotomies and "other" procedures. Within the category of brain tumour surgery, there may be children who are at much higher risk for POV, balanced out by those at lower risk. A secondary analysis of these data for children with posterior fossa brain tumours, in particular, would be useful.

That the oldest age quartile (12 to <17 years) emerged as a protective factor for POV by 120 hours, once intraoperative variables were controlled for, is consistent with research in children following other types of surgery [27], but has not been supported in research for POV after craniotomy [11]. The two middle quartiles (ages 4 to <12 years) were more likely to vomit earlier, but their odds of vomiting were no different than those under four years of age by 120 hours. Infants and young children in this group of children may present later with vomiting and "catch-up" to school aged children by 120 hours. This result points to the importance of examining POV beyond the first 24 hours and continuing prophylactic use of anti-emetics beyond 24 hours in at-risk groups. Interestingly, gender was not shown to be a significant risk factor and, therefore, an interaction effect of gender and age was not examined, despite the idea that females after puberty may be at higher risk for POV [27].

While not desflurane was commonly used in this sample, any use of desflurane was identified as a risk factor for early vomiting compared to the use of other volatile anaesthetics. This finding must be taken in the context of a small sample size and very wide confidence intervals when all other variables in the model were controlled for. The Stollery Children's Hospital site had a greater proportion of exposed children than the Hospital for Sick Children and the influence of individual clinicians was not accounted for. As previously discussed, the use of desflurane has been identified as a significant risk factor when compared to other volatile anaesthetics in a multivariable analysis of risk factors for PONV for adults requiring microvascular decompression of cranial nerves [20]; this is why we chose to include it, and compare it to a grouping of other volatile anaesthetics (isoflurane, sevoflurane and combinations of these) in the multivariable analysis. The use of desflurane in children has the advantage of rapid recovery [28] which must be clinically balanced with any potential disadvantages in this clinical population if chosen; however, its association with POV in this study warrants reconsideration of its use when there are other efficacious anesthetic options.

Children who received intraoperative ondansetron were more likely to vomit by 120 hours those who did not. This finding may be due to use of clinical judgment in administering ondansetron, a drug which has not been shown to have efficacy in preventing vomiting in children after craniotomy [6,11]. Thus, the administration of the drug was predictive of vomiting due to its administration to those correctly judged likely to vomit due to influences not measured or included in the multivariable analysis. Apfel and colleagues [4] also did not find a significant effect for intraoperative anti-emetics in decreasing POV in their multivariable logistic regression models. Interesting is the significant moderating effect of the intraoperative ondansetron on the use of desflurane as shown by the significant interaction term in the first multivariable model. Future studies of the efficacy of ondansetron, and other 5-HT_3 _receptor antagonists, in preventing POV in this group of children might then include stratification for characteristics of the volatile anaesthetic used.

The intraoperative use of dexamethasone was not shown to be a protective factor for POV by 120 hours. Like ondansetron, its use may have been targeted to children clinically perceived to be at high risk for POV, and so a protective effect might not emerge in a retrospective study. The lack of significance of the use of intraoperative dexamethasone and the lack of an interaction effect between dexamethasone and ondansetron may also be reflective of this limitation. Additionally, we did not control for the use of preoperative dexamethasone because preoperative dexamethasone was given to most of the children with brain tumours, and thus collinearity with the type of surgery would be a problem. However, Subramaniam and colleagues [11] did look at preoperative dexamethasone treatment in children with brain tumours in their randomized controlled trial of ondansetron. These authors found no difference in PONV between children who received dexamethasone preoperatively and those who did not. Length of surgery as a continuous variable was also not a significant risk factor for POV by 120 hours in this sample, which is consistent with previous research, where the cut off for a protective effect for length of surgery was 30 minutes [3,9].

Finally, associations between POV by 120 hours and a number of negative consequences for recovery – the development of a pseudomeningocele, infection, wound failure/cerebral spinal fluid leak, and posterior fossa syndrome (in children with brain tumours) – could not be identified in the data. These outcomes may be better studied prospectively with consideration of the severity of POV. A prospective study would also aid in identifying a causal pathway in the relationship between negative outcomes and POV.

The primary limitation of this study is its retrospective nature as it limits the researchers' control over the data that can be collected. For the outcomes of nausea, vomiting, and retching, the quality of the charting by health care professionals was paramount. The exact count of vomiting episodes was likely underestimated, as severe events were often identified as "+ + + vomiting," but the cumulative incidence of vomiting at the specified time period should be accurate. Additionally, retching may have only been charted if it occurred without vomiting, for example in the post-anaesthesia recovery room or paediatric intensive care. Once the child was cared for in the general nursing care unit, it may have been unobserved by the health care team and thus not documented. Subjective measures of nausea, expressed by the child, were rarely charted. In adults after craniotomy, risk factors for nausea may be different than those for vomiting [7]. As we were unable to evaluate risk and protective factors for nausea, the results of this study can not be applied to PON.

There were also challenges in conducting the study at two sites. Differences in the way that postoperative neurosurgical care was provided and the timing of transfer of the child either home, for rehabilitation, or for oncology treatment, varied between institutions. Different documentation styles and charting practices may have also affected data collection. Defining what constituted the acute postoperative period was also difficult and the outcomes for analysis of risk and protective factors were decided once the data were collected and descriptive statistics completed.

Sample size issues also became apparent in the final stages of the multivariable analysis, as shown by the wide confidence intervals for some variables. Initially we had estimated a sample size of approximately 300. There were more children with exclusion criteria than expected, which decreased the final sample. The high incidence of vomiting, even when the outcome was limited to POV by 120 hours, resulted in five children without vomiting per variable for the final multivariable model. Therefore, the results of the multivariable analysis should be interpreted with caution and require further validation.

Due to the retrospective nature of the data collection, the multivariable models developed in this study can be used to identify risk and protective factors for POV in children after posterior fossa surgery in general. The models were not developed for prognosis or risk scoring at the individual level. The study does not show the effect of treatments, only their contribution as possible risk or protective factors. The type of data that can be extracted is limited to what is charted in a retrospective study. For example, whether or not the administration of a postoperative anti-emetic was prophylactic or therapeutic could not be reliably determined and therefore was not used in the analysis. A further limitation of the study is that the outcomes examined for the risk and protective factors were one or more events of POV by 120 hours and early vs. late POV. Severity of POV, an important outcome that is often clinically observed in children after posterior fossa surgery, and may be inferred from the frequency of events and length of time that POV was experienced, was not quantified for the analysis of risk and protective factors.

## Conclusion

The findings of risk and protective factors in this study support the suggestion that current prognostic models and risk scoring systems for POV should not be used in children after posterior fossa craniotomy [29] as they may be different for this population. Given the descriptive findings of how common POV is in this group of children after posterior fossa surgery, guidelines such as those proposed by The Society for Ambulatory Anesthesia [30] for POV in high risk populations should be considered. These guidelines include the use of two or three prophylactic drugs from different classes for children who are at high risk for POV and to consider the use of total intravenous anaesthesia (TIVA). With established POV, drugs from another class than that already in use ought to be considered. Importantly, in a Bayesian Meta-analysis of six single drugs and five combinations, Engelman, Salengaros, and Barvais [31] found the greatest relative risk reduction (80%) in POV with the combination of the 5-HT_3 _receptor antagonists and dexamethasone. Of particular note, is the current absence of evidence that any class of drug, including the combination of dexamethasone and ondansetron, is effective for preventing or treating POV in children after craniotomy. Overall, this is a unique but understudied population of children who are at risk for POV.

There have also not been any studies of particular anaesthetic protocols, such as TIVA with propofol, in children requiring posterior fossa surgery. Knowledge and treatment guidelines are currently limited to what is known in other patient populations. Translating current knowledge and treatment guidelines to children requiring posterior fossa surgery is challenging. For example, even the results of studies of TIVA in adults after craniotomy remain inconclusive [32]. Finally, given the length of time that children experience POV, it is necessary that protocols for inpatient anti-emetic care, including length of time postoperative dexamethasone is administered and the postoperative use of intermittent use of anti-emetics, be developed and tested in children requiring posterior fossa craniotomy. Ultimately, a methodical approach to preventing and treating POV, starting in the preoperative period, following through the child's course of care in the hospital and included in discharge planning, is required for this vulnerable group of children.

## Competing interests

The authors declare that they have no competing interests.

## Authors' contributions

SN Conceptualized the study, collected data at the Stollery Children's Hospital Site, entered and analyzed the data, and drafted the manuscript, revised and prepared the manuscript for publication. CNC Helped with the study design and methodology, critically revised the manuscript and approved the final version for publication. DS Developed the statistical plan for the manuscript, directed the statistical analysis and interpretation of the data, and critically revised the statistical portion of the manuscript. BD Helped with the conceptualization of the study, collected data at the Hospital for Sick Children, critically revised the manuscript and reviewed the final version for publication. HY Helped conceptualize the study, assisted in the development of the data collection tool, and was study coordinator at the Hospital for Sick Children. JD Helped with conceptualization of the study, critically revised the manuscript, and approved the final version for publication. All authors read and approved the final version of the manuscript.

## Authors' information

SM, BD, and HY are nurses who work in children's neurosurgery, where their clinical experience prompted them to identify the extent of postoperative nausea and vomiting on their units, not only to gain knowledge of the problem but to then identify ways to help these children and their families.

## Pre-publication history

The pre-publication history for this paper can be accessed here:


